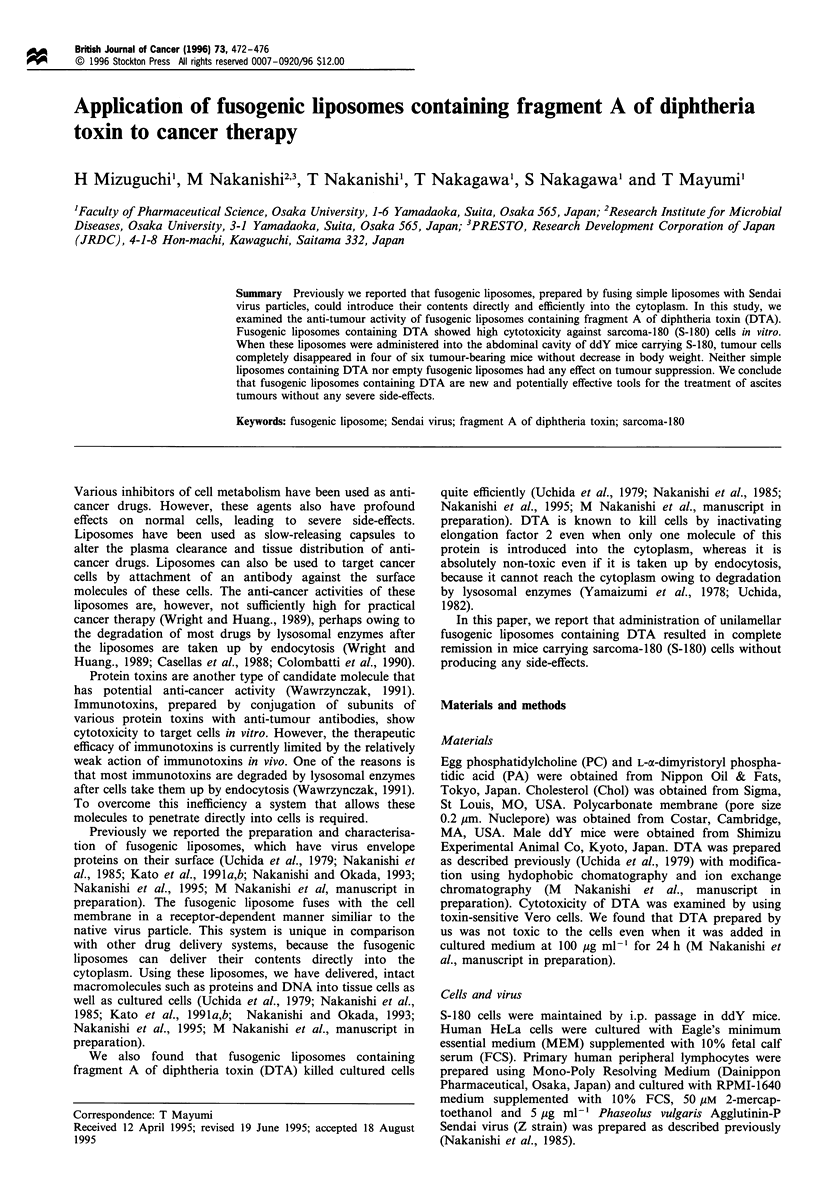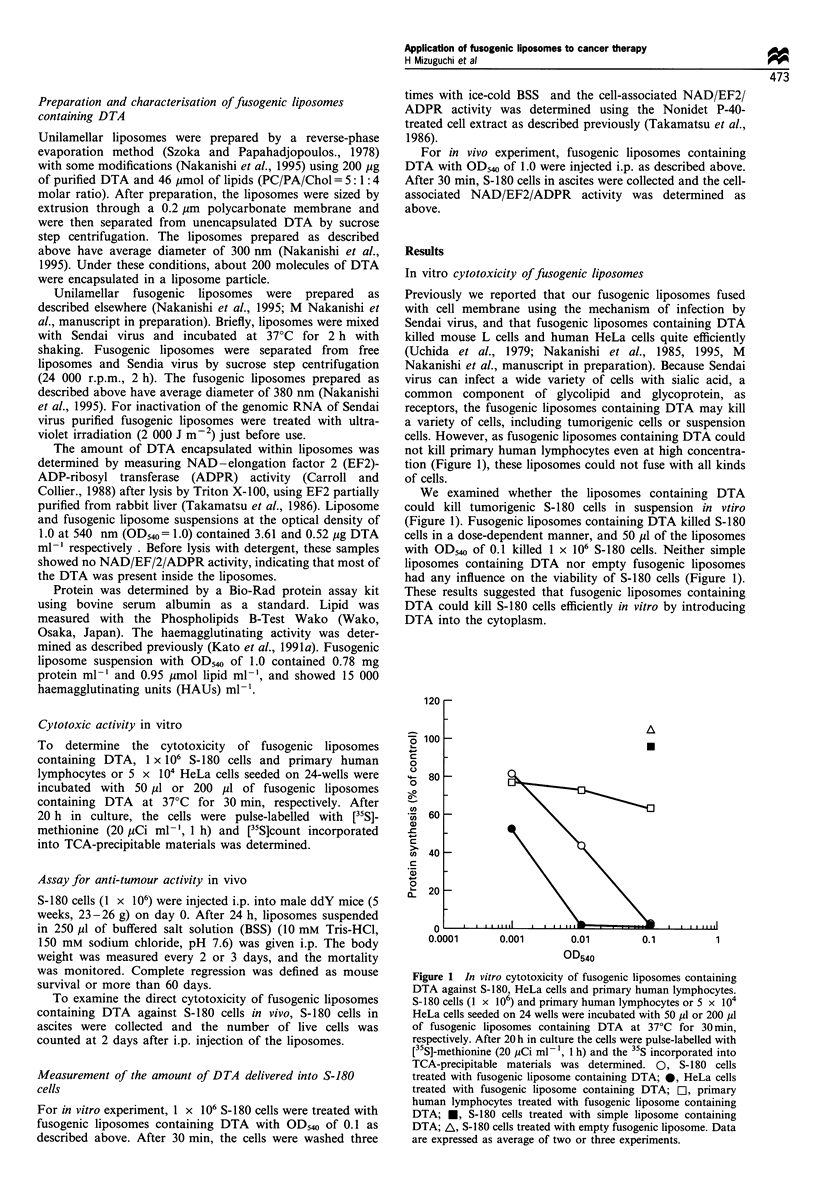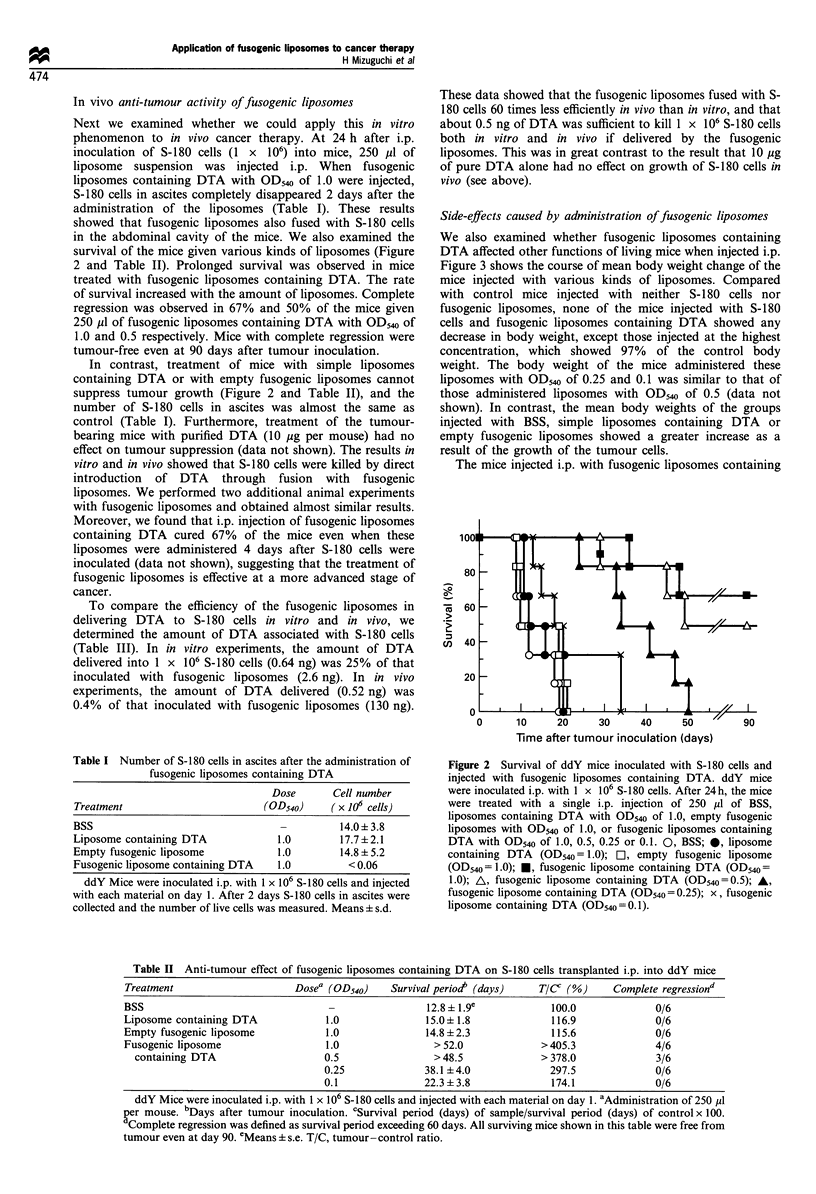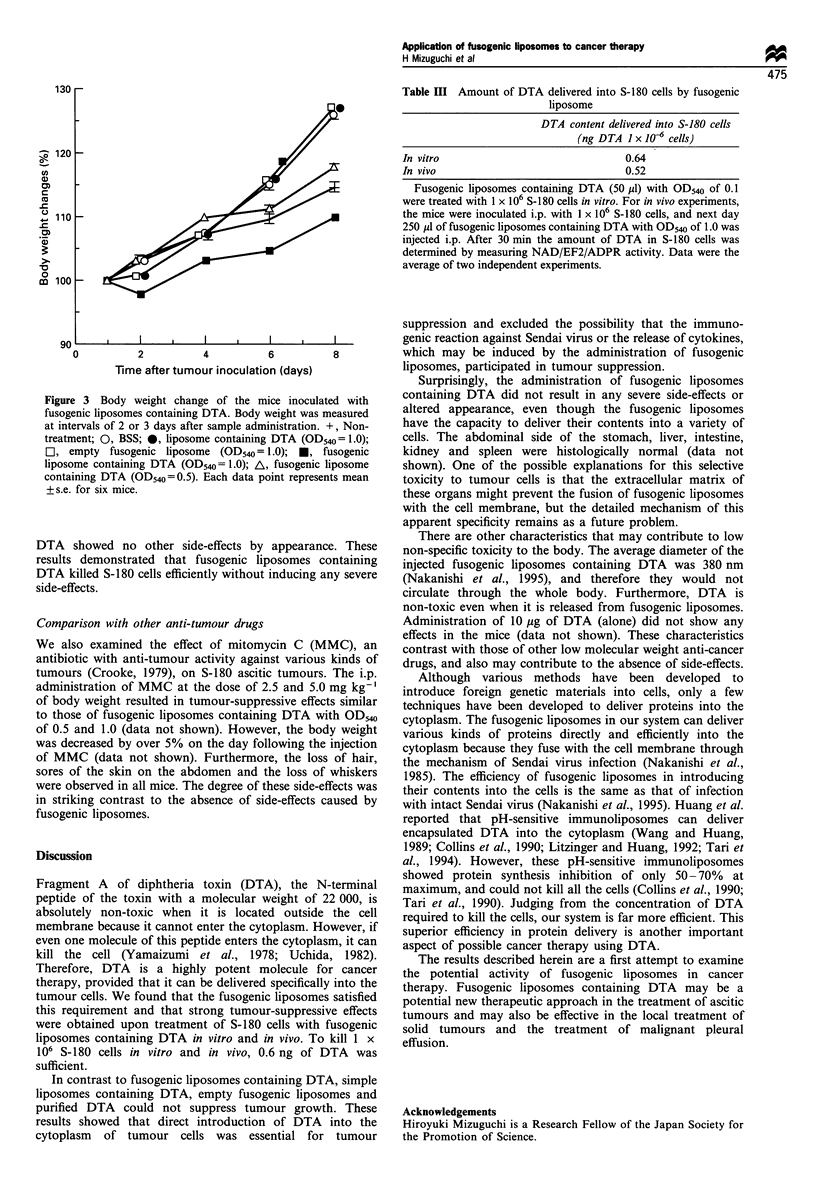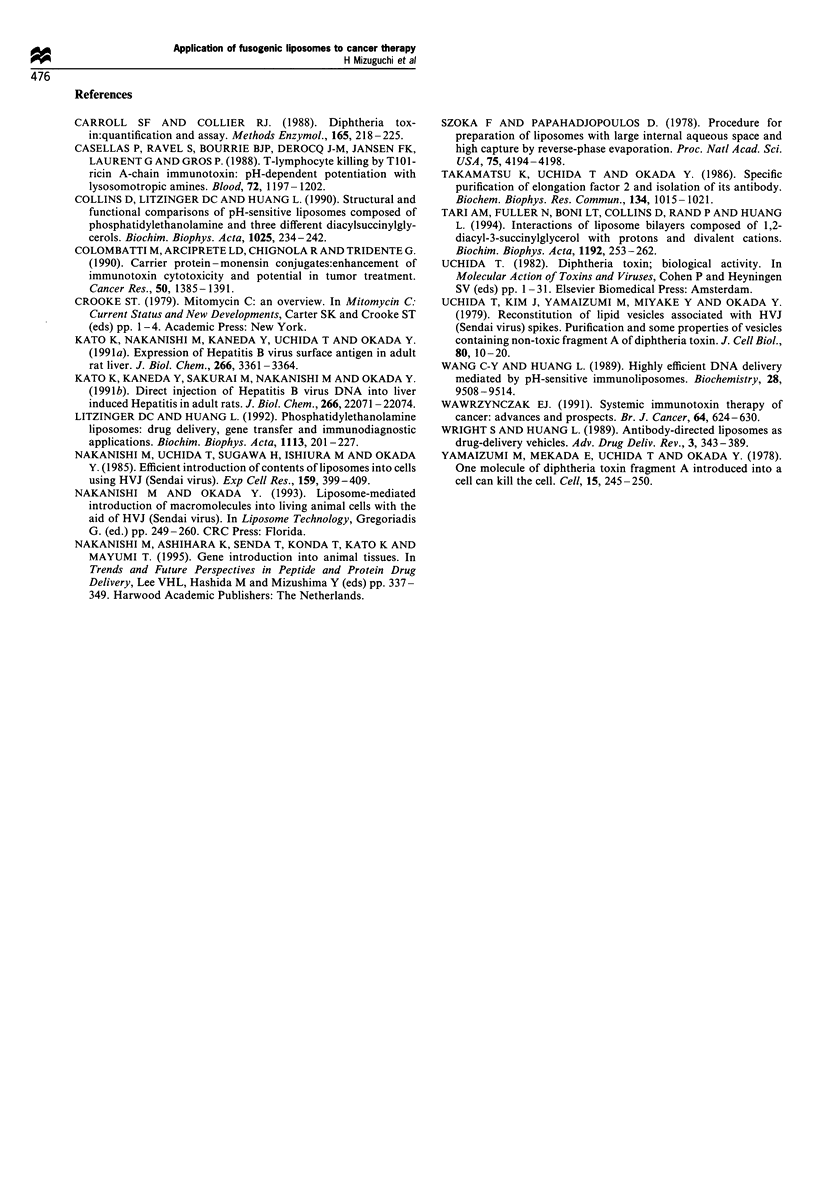# Application of fusogenic liposomes containing fragment A of diphtheria toxin to cancer therapy.

**DOI:** 10.1038/bjc.1996.83

**Published:** 1996-02

**Authors:** H. Mizuguchi, M. Nakanishi, T. Nakanishi, T. Nakagawa, S. Nakagawa, T. Mayumi

**Affiliations:** Faculty of Pharmaceutical Science, Osaka University, Japan.

## Abstract

Previously we reported that fusogenic liposomes, prepared by fusing simple liposomes with Sendai virus particles, could introduce their contents directly and efficiently into the cytoplasm. In this study, we examined the anti-tumour activity of fusogenic liposomes containing fragment A of diphtheria toxin (DTA). Fusogenic liposomes containing DTA showed high cytotoxicity against sarcoma-180 (S-180) cells in vitro. When these liposomes were administered into the abdominal cavity of ddY mice carrying S-180, tumour cells completely disappeared in four of six tumour-bearing mice without decrease in body weight. Neither simple liposomes containing DTA nor empty fusogenic liposomes had any effect on tumour suppression. We conclude that fusogenic liposomes containing DTA are new and potentially effective tools for the treatment of ascites tumours without any severe side-effects.


British Journal of Cancer (1996) 73, 472-476
© 1996 Stockton Press All rights reserved 0007-0920/96 $12.00

### Application of fusogenic liposomes containing fragment A of diphtheria toxin to cancer therapy

H Mizuguchi[1], M Nakanishi[2,3], T Nakanishi[1], T Nakagawa[1], S Nakagawa[1] and T Mayumi[1]

[1]*Faculty of Pharmaceutical Science, Osaka University, 1-6 Yamadaoka, Suita, Osaka 565, Japan;* [2]*Research Institute for Microbial Diseases, Osaka University, 3-1 Yamadaoka, Suita, Osaka 565, Japan;* [3]*PRESTO, Research Development Corporation of Japan (JRDC), 4-1-8 Hon-machi, Kawaguchi, Saitama 332, Japan*

**Summary** Previously we reported that fusogenic liposomes, prepared by fusing simple liposomes with Sendai virus particles, could introduce their contents directly and efficiently into the cytoplasm. In this study, we examined the anti-tumour activity of fusogenic liposomes containing fragment A of diphtheria toxin (DTA). Fusogenic liposomes containing DTA showed high cytotoxicity against sarcoma-180 (S-180) cells *in vitro*. When these liposomes were administered into the abdominal cavity of ddY mice carrying S-180, tumour cells completely disappeared in four of six tumour-bearing mice without decrease in body weight. Neither simple liposomes containing DTA nor empty fusogenic liposomes had any effect on tumour suppression. We conclude that fusogenic liposomes containing DTA are new and potentially effective tools for the treatment of ascites tumours without any severe side-effects.

**Keywords:** fusogenic liposome; Sendai virus; fragment A of diphtheria toxin; sarcoma-180

Various inhibitors of cell metabolism have been used as anti-cancer drugs. However, these agents also have profound effects on normal cells, leading to severe side-effects. Liposomes have been used as slow-releasing capsules to alter the plasma clearance and tissue distribution of anti-cancer drugs. Liposomes can also be used to target cancer cells by attachment of an antibody against the surface molecules of these cells. The anti-cancer activities of these liposomes are, however, not sufficiently high for practical cancer therapy (Wright and Huang., 1989), perhaps owing to the degradation of most drugs by lysosomal enzymes after the liposomes are taken up by endocytosis (Wright and Huang., 1989; Casellas *et al.*, 1988; Colombatti *et al.*, 1990).

Protein toxins are another type of candidate molecule that has potential anti-cancer activity (Wawrzynczak, 1991). Immunotoxins, prepared by conjugation of subunits of various protein toxins with anti-tumour antibodies, show cytotoxicity to target cells *in vitro*. However, the therapeutic efficacy of immunotoxins is currently limited by the relatively weak action of immunotoxins *in vivo*. One of the reasons is that most immunotoxins are degraded by lysosomal enzymes after cells take them up by endocytosis (Wawrzynczak, 1991). To overcome this inefficiency a system that allows these molecules to penetrate directly into cells is required.

Previously we reported the preparation and characterisation of fusogenic liposomes, which have virus envelope proteins on their surface (Uchida *et al.*, 1979; Nakanishi *et al.*, 1985; Kato *et al.*, 1991*a,b*; Nakanishi and Okada, 1993; Nakanishi *et al.*, 1995; M Nakanishi *et al*, manuscript in preparation). The fusogenic liposome fuses with the cell membrane in a receptor-dependent manner similiar to the native virus particle. This system is unique in comparison with other drug delivery systems, because the fusogenic liposomes can deliver their contents directly into the cytoplasm. Using these liposomes, we have delivered, intact macromolecules such as proteins and DNA into tissue cells as well as cultured cells (Uchida *et al.*, 1979; Nakanishi *et al.*, 1985; Kato *et al.*, 1991*a,b*; Nakanishi and Okada, 1993; Nakanishi *et al.*, 1995; M Nakanishi *et al.*, manuscript in preparation).

We also found that fusogenic liposomes containing fragment A of diphtheria toxin (DTA) killed cultured cells quite efficiently (Uchida *et al.*, 1979; Nakanishi *et al.*, 1985; Nakanishi *et al.*, 1995; M Nakanishi *et al.*, manuscript in preparation). DTA is known to kill cells by inactivating elongation factor 2 even when only one molecule of this protein is introduced into the cytoplasm, whereas it is absolutely non-toxic even if it is taken up by endocytosis, because it cannot reach the cytoplasm owing to degradation by lysosomal enzymes (Yamaizumi *et al.*, 1978; Uchida, 1982).

In this paper, we report that administration of unilamellar fusogenic liposomes containing DTA resulted in complete remission in mice carrying sarcoma-180 (S-180) cells without producing any side-effects.

### Materials and methods

### *Materials*

Egg phosphatidylcholine (PC) and L-α-dimyristoryl phosphatidic acid (PA) were obtained from Nippon Oil & Fats, Tokyo, Japan. Cholesterol (Chol) was obtained from Sigma, St Louis, MO, USA. Polycarbonate membrane (pore size 0.2 μm. Nuclepore) was obtained from Costar, Cambridge, MA, USA. Male ddY mice were obtained from Shimizu Experimental Animal Co, Kyoto, Japan. DTA was prepared as described previously (Uchida *et al.*, 1979) with modification using hydophobic chomatography and ion exchange chromatography (M Nakanishi *et al.*, manuscript in preparation). Cytotoxicity of DTA was examined by using toxin-sensitive Vero cells. We found that DTA prepared by us was not toxic to the cells even when it was added in cultured medium at 100 $\mu g\ ml^{-1}$ for 24 h (M Nakanishi *et al.*, manuscript in preparation).

### *Cells and virus*

S-180 cells were maintained by i.p. passage in ddY mice. Human HeLa cells were cultured with Eagle's minimum essential medium (MEM) supplemented with 10% fetal calf serum (FCS). Primary human peripheral lymphocytes were prepared using Mono-Poly Resolving Medium (Dainippon Pharmaceutical, Osaka, Japan) and cultured with RPMI-1640 medium supplemented with 10% FCS, 50 μM 2-mercaptoethanol and 5 $\mu g\ ml^{-1}$ *Phaseolus vulgaris* Agglutinin-P Sendai virus (Z strain) was prepared as described previously (Nakanishi *et al.*, 1985).

Correspondence: T Mayumi
Received 12 April 1995; revised 19 June 1995; accepted 18 August 1995

*Preparation and characterisation of fusogenic liposomes containing DTA*

Unilamellar liposomes were prepared by a reverse-phase evaporation method (Szoka and Papahadjopoulos., 1978) with some modifications (Nakanishi *et al.*, 1995) using 200 μg of purified DTA and 46 μmol of lipids (PC/PA/Chol = 5:1:4 molar ratio). After preparation, the liposomes were sized by extrusion through a 0.2 μm polycarbonate membrane and were then separated from unencapsulated DTA by sucrose step centrifugation. The liposomes prepared as described above have average diameter of 300 nm (Nakanishi *et al.*, 1995). Under these conditions, about 200 molecules of DTA were encapsulated in a liposome particle.

Unilamellar fusogenic liposomes were prepared as described elsewhere (Nakanishi *et al.*, 1995; M Nakanishi *et al.*, manuscript in preparation). Briefly, liposomes were mixed with Sendai virus and incubated at 37°C for 2 h with shaking. Fusogenic liposomes were separated from free liposomes and Sendia virus by sucrose step centrifugation (24 000 r.p.m., 2 h). The fusogenic liposomes prepared as described above have average diameter of 380 nm (Nakanishi *et al.*, 1995). For inactivation of the genomic RNA of Sendai virus purified fusogenic liposomes were treated with ultraviolet irradiation (2 000 J $m^{-2}$) just before use.

The amount of DTA encapsulated within liposomes was determined by measuring NAD–elongation factor 2 (EF2)-ADP-ribosyl transferase (ADPR) activity (Carroll and Collier., 1988) after lysis by Triton X-100, using EF2 partially purified from rabbit liver (Takamatsu *et al.*, 1986). Liposome and fusogenic liposome suspensions at the optical density of 1.0 at 540 nm ($OD_{540}$ = 1.0) contained 3.61 and 0.52 μg DTA $ml^{-1}$ respectively . Before lysis with detergent, these samples showed no NAD/EF/2/ADPR activity, indicating that most of the DTA was present inside the liposomes.

Protein was determined by a Bio-Rad protein assay kit using bovine serum albumin as a standard. Lipid was measured with the Phospholipids B-Test Wako (Wako, Osaka, Japan). The haemagglutinating activity was determined as described previously (Kato *et al.*, 1991*a*). Fusogenic liposome suspension with $OD_{540}$ of 1.0 contained 0.78 mg protein $ml^{-1}$ and 0.95 μmol lipid $ml^{-1}$, and showed 15 000 haemagglutinating units (HAUs) $ml^{-1}$.

*Cytotoxic activity* in vitro

To determine the cytotoxicity of fusogenic liposomes containing DTA, $1 \times 10^6$ S-180 cells and primary human lymphocytes or $5 \times 10^4$ HeLa cells seeded on 24-wells were incubated with 50 μl or 200 μl of fusogenic liposomes containing DTA at 37°C for 30 min, respectively. After 20 h in culture, the cells were pulse-labelled with [$^{35}$S]-methionine (20 μCi $ml^{-1}$, 1 h) and [$^{35}$S]count incorporated into TCA-precipitable materials was determined.

*Assay for anti-tumour activity* in vivo

S-180 cells ($1 \times 10^6$) were injected i.p. into male ddY mice (5 weeks, 23–26 g) on day 0. After 24 h, liposomes suspended in 250 μl of buffered salt solution (BSS) (10 mM Tris-HCl, 150 mM sodium chloride, pH 7.6) was given i.p. The body weight was measured every 2 or 3 days, and the mortality was monitored. Complete regression was defined as mouse survival or more than 60 days.

To examine the direct cytotoxicity of fusogenic liposomes containing DTA against S-180 cells *in vivo*, S-180 cells in ascites were collected and the number of live cells was counted at 2 days after i.p. injection of the liposomes.

*Measurement of the amount of DTA delivered into S-180 cells*

For *in vitro* experiment, $1 \times 10^6$ S-180 cells were treated with fusogenic liposomes containing DTA with $OD_{540}$ of 0.1 as described above. After 30 min, the cells were washed three times with ice-cold BSS and the cell-associated NAD/EF2/ADPR activity was determined using the Nonidet P-40-treated cell extract as described previously (Takamatsu *et al.*, 1986).

For *in vivo* experiment, fusogenic liposomes containing DTA with $OD_{540}$ of 1.0 were injected i.p. as described above. After 30 min, S-180 cells in ascites were collected and the cell-associated NAD/EF2/ADPR activity was determined as above.

### Results

In vitro *cytotoxicity of fusogenic liposomes*

Previously we reported that our fusogenic liposomes fused with cell membrane using the mechanism of infection by Sendai virus, and that fusogenic liposomes containing DTA killed mouse L cells and human HeLa cells quite efficiently (Uchida *et al.*, 1979; Nakanishi *et al.*, 1985, 1995, M Nakanishi *et al.*, manuscript in preparation). Because Sendai virus can infect a wide variety of cells with sialic acid, a common component of glycolipid and glycoprotein, as receptors, the fusogenic liposomes containing DTA may kill a variety of cells, including tumorigenic cells or suspension cells. However, as fusogenic liposomes containing DTA could not kill primary human lymphocytes even at high concentration (Figure 1), these liposomes could not fuse with all kinds of cells.

We examined whether the liposomes containing DTA could kill tumorigenic S-180 cells in suspension *in vtiro* (Figure 1). Fusogenic liposomes containing DTA killed S-180 cells in a dose-dependent manner, and 50 μl of the liposomes with $OD_{540}$ of 0.1 killed $1 \times 10^6$ S-180 cells. Neither simple liposomes containing DTA nor empty fusogenic liposomes had any influence on the viability of S-180 cells (Figure 1). These results suggested that fusogenic liposomes containing DTA could kill S-180 cells efficiently *in vitro* by introducing DTA into the cytoplasm.

**Figure 1** *In vitro* cytotoxicity of fusogenic liposomes containing DTA against S-180, HeLa cells and primary human lymphocytes. S-180 cells ($1 \times 10^6$) and primary human lymphocytes or $5 \times 10^4$ HeLa cells seeded on 24 wells were incubated with 50 μl or 200 μl of fusogenic liposomes containing DTA at 37°C for 30 min, respectively. After 20 h in culture the cells were pulse-labelled with [$^{35}$S]-methionine (20 μCi $ml^{-1}$, 1 h) and the $^{35}$S incorporated into TCA-precipitable materials was determined. ○, S-180 cells treated with fusogenic liposome containing DTA; ●, HeLa cells treated with fusogenic liposome containing DTA; □, primary human lymphocytes treated with fusogenic liposome containing DTA; ■, S-180 cells treated with simple liposome containing DTA; △, S-180 cells treated with empty fusogenic liposome. Data are expressed as average of two or three experiments.

### In vivo *anti-tumour activity of fusogenic liposomes*

Next we examined whether we could apply this *in vitro* phenomenon to *in vivo* cancer therapy. At 24 h after i.p. inoculation of S-180 cells ($1 \times 10^6$) into mice, 250 $\mu$l of liposome suspension was injected i.p. When fusogenic liposomes containing DTA with $OD_{540}$ of 1.0 were injected, S-180 cells in ascites completely disappeared 2 days after the administration of the liposomes (Table I). These results showed that fusogenic liposomes also fused with S-180 cells in the abdominal cavity of the mice. We also examined the survival of the mice given various kinds of liposomes (Figure 2 and Table II). Prolonged survival was observed in mice treated with fusogenic liposomes containing DTA. The rate of survival increased with the amount of liposomes. Complete regression was observed in 67% and 50% of the mice given 250 $\mu$l of fusogenic liposomes containing DTA with $OD_{540}$ of 1.0 and 0.5 respectively. Mice with complete regression were tumour-free even at 90 days after tumour inoculation.

In contrast, treatment of mice with simple liposomes containing DTA or with empty fusogenic liposomes cannot suppress tumour growth (Figure 2 and Table II), and the number of S-180 cells in ascites was almost the same as control (Table I). Furthermore, treatment of the tumour-bearing mice with purified DTA (10 $\mu$g per mouse) had no effect on tumour suppression (data not shown). The results *in vitro* and *in vivo* showed that S-180 cells were killed by direct introduction of DTA through fusion with fusogenic liposomes. We performed two additional animal experiments with fusogenic liposomes and obtained almost similar results. Moreover, we found that i.p. injection of fusogenic liposomes containing DTA cured 67% of the mice even when these liposomes were administered 4 days after S-180 cells were inoculated (data not shown), suggesting that the treatment of fusogenic liposomes is effective at a more advanced stage of cancer.

To compare the efficiency of the fusogenic liposomes in delivering DTA to S-180 cells *in vitro* and *in vivo*, we determined the amount of DTA associated with S-180 cells (Table III). In *in vitro* experiments, the amount of DTA delivered into $1 \times 10^6$ S-180 cells (0.64 ng) was 25% of that inoculated with fusogenic liposomes (2.6 ng). In *in vivo* experiments, the amount of DTA delivered (0.52 ng) was 0.4% of that inoculated with fusogenic liposomes (130 ng).

**Table I** Number of S-180 cells in ascites after the administration of fusogenic liposomes containing DTA

| *Treatment* | *Dose ($OD_{540}$)* | *Cell number ($\times 10^6$ cells)* |
|---|---|---|
| BSS | – | 14.0±3.8 |
| Liposome containing DTA | 1.0 | 17.7±2.1 |
| Empty fusogenic liposome | 1.0 | 14.8±5.2 |
| Fusogenic liposome containing DTA | 1.0 | <0.06 |

ddY Mice were inoculated i.p. with $1 \times 10^6$ S-180 cells and injected with each material on day 1. After 2 days S-180 cells in ascites were collected and the number of live cells was measured. Means±s.d.

These data showed that the fusogenic liposomes fused with S-180 cells 60 times less efficiently *in vivo* than *in vitro*, and that about 0.5 ng of DTA was sufficient to kill $1 \times 10^6$ S-180 cells both *in vitro* and *in vivo* if delivered by the fusogenic liposomes. This was in great contrast to the result that 10 $\mu$g of pure DTA alone had no effect on growth of S-180 cells *in vivo* (see above).

### *Side-effects caused by administration of fusogenic liposomes*

We also examined whether fusogenic liposomes containing DTA affected other functions of living mice when injected i.p. Figure 3 shows the course of mean body weight change of the mice injected with various kinds of liposomes. Compared with control mice injected with neither S-180 cells nor fusogenic liposomes, none of the mice injected with S-180 cells and fusogenic liposomes containing DTA showed any decrease in body weight, except those injected at the highest concentration, which showed 97% of the control body weight. The body weight of the mice administered these liposomes with $OD_{540}$ of 0.25 and 0.1 was similar to that of those administered liposomes with $OD_{540}$ of 0.5 (data not shown). In contrast, the mean body weights of the groups injected with BSS, simple liposomes containing DTA or empty fusogenic liposomes showed a greater increase as a result of the growth of the tumour cells.

The mice injected i.p. with fusogenic liposomes containing

**Figure 2** Survival of ddY mice inoculated with S-180 cells and injected with fusogenic liposomes containing DTA. ddY mice were inoculated i.p. with $1 \times 10^6$ S-180 cells. After 24 h, the mice were treated with a single i.p. injection of 250 $\mu$l of BSS, liposomes containing DTA with $OD_{540}$ of 1.0, empty fusogenic liposomes with $OD_{540}$ of 1.0, or fusogenic liposomes containing DTA with $OD_{540}$ of 1.0, 0.5, 0.25 or 0.1. ○, BSS; ●, liposome containing DTA ($OD_{540}$=1.0); □, empty fusogenic liposome ($OD_{540}$=1.0); ■, fusogenic liposome containing DTA ($OD_{540}$=1.0); △, fusogenic liposome containing DTA ($OD_{540}$=0.5); ▲, fusogenic liposome containing DTA ($OD_{540}$=0.25); ×, fusogenic liposome containing DTA ($OD_{540}$=0.1).

**Table II** Anti-tumour effect of fusogenic liposomes containing DTA on S-180 cells transplanted i.p. into ddY mice

| *Treatment* | *Dose[a] ($OD_{540}$)* | *Survival period[b] (days)* | *T/C[c] (%)* | *Complete regression[d]* |
|---|---|---|---|---|
| BSS | – | 12.8±1.9[e] | 100.0 | 0/6 |
| Liposome containing DTA | 1.0 | 15.0±1.8 | 116.9 | 0/6 |
| Empty fusogenic liposome | 1.0 | 14.8±2.3 | 115.6 | 0/6 |
| Fusogenic liposome | 1.0 | >52.0 | >405.3 | 4/6 |
| containing DTA | 0.5 | >48.5 | >378.0 | 3/6 |
| | 0.25 | 38.1±4.0 | 297.5 | 0/6 |
| | 0.1 | 22.3±3.8 | 174.1 | 0/6 |

ddY Mice were inoculated i.p. with $1 \times 10^6$ S-180 cells and injected with each material on day 1. [a]Administration of 250 $\mu$l per mouse. [b]Days after tumour inoculation. [c]Survival period (days) of sample/survival period (days) of control $\times$ 100. [d]Complete regression was defined as survival period exceeding 60 days. All surviving mice shown in this table were free from tumour even at day 90. [e]Means±s.e. T/C, tumour–control ratio.

**Figure 3** Body weight change of the mice inoculated with fusogenic liposomes containing DTA. Body weight was measured at intervals of 2 or 3 days after sample administration. +, Non-treatment; ○, BSS; ●, liposome containing DTA ($OD_{540}=1.0$); □, empty fusogenic liposome ($OD_{540}=1.0$); ■, fusogenic liposome containing DTA ($OD_{540}=1.0$); △, fusogenic liposome containing DTA ($OD_{540}=0.5$). Each data point represents mean ±s.e. for six mice.

DTA showed no other side-effects by appearance. These results demonstrated that fusogenic liposomes containing DTA killed S-180 cells efficiently without inducing any severe side-effects.

*Comparison with other anti-tumour drugs*

We also examined the effect of mitomycin C (MMC), an antibiotic with anti-tumour activity against various kinds of tumours (Crooke, 1979), on S-180 ascitic tumours. The i.p. administration of MMC at the dose of 2.5 and 5.0 mg $kg^{-1}$ of body weight resulted in tumour-suppressive effects similar to those of fusogenic liposomes containing DTA with $OD_{540}$ of 0.5 and 1.0 (data not shown). However, the body weight was decreased by over 5% on the day following the injection of MMC (data not shown). Furthermore, the loss of hair, sores of the skin on the abdomen and the loss of whiskers were observed in all mice. The degree of these side-effects was in striking contrast to the absence of side-effects caused by fusogenic liposomes.

### Discussion

Fragment A of diphtheria toxin (DTA), the N-terminal peptide of the toxin with a molecular weight of 22 000, is absolutely non-toxic when it is located outside the cell membrane because it cannot enter the cytoplasm. However, if even one molecule of this peptide enters the cytoplasm, it can kill the cell (Yamaizumi *et al.*, 1978; Uchida, 1982). Therefore, DTA is a highly potent molecule for cancer therapy, provided that it can be delivered specifically into the tumour cells. We found that the fusogenic liposomes satisfied this requirement and that strong tumour-suppressive effects were obtained upon treatment of S-180 cells with fusogenic liposomes containing DTA *in vitro* and *in vivo*. To kill 1 × $10^6$ S-180 cells *in vitro* and *in vivo*, 0.6 ng of DTA was sufficient.

In contrast to fusogenic liposomes containing DTA, simple liposomes containing DTA, empty fusogenic liposomes and purified DTA could not suppress tumour growth. These results showed that direct introduction of DTA into the cytoplasm of tumour cells was essential for tumour

**Table III** Amount of DTA delivered into S-180 cells by fusogenic liposome

| | *DTA content delivered into S-180 cells (ng DTA 1 × $10^{-6}$ cells)* |
|---|---|
| *In vitro* | 0.64 |
| *In vivo* | 0.52 |

Fusogenic liposomes containing DTA (50 μl) with $OD_{540}$ of 0.1 were treated with 1 × $10^6$ S-180 cells *in vitro*. For *in vivo* experiments, the mice were inoculated i.p. with 1 × $10^6$ S-180 cells, and next day 250 μl of fusogenic liposomes containing DTA with $OD_{540}$ of 1.0 was injected i.p. After 30 min the amount of DTA in S-180 cells was determined by measuring NAD/EF2/ADPR activity. Data were the average of two independent experiments.

suppression and excluded the possibility that the immunogenic reaction against Sendai virus or the release of cytokines, which may be induced by the administration of fusogenic liposomes, participated in tumour suppression.

Surprisingly, the administration of fusogenic liposomes containing DTA did not result in any severe side-effects or altered appearance, even though the fusogenic liposomes have the capacity to deliver their contents into a variety of cells. The abdominal side of the stomach, liver, intestine, kidney and spleen were histologically normal (data not shown). One of the possible explanations for this selective toxicity to tumour cells is that the extracellular matrix of these organs might prevent the fusion of fusogenic liposomes with the cell membrane, but the detailed mechanism of this apparent specificity remains as a future problem.

There are other characteristics that may contribute to low non-specific toxicity to the body. The average diameter of the injected fusogenic liposomes containing DTA was 380 nm (Nakanishi *et al.*, 1995), and therefore they would not circulate through the whole body. Furthermore, DTA is non-toxic even when it is released from fusogenic liposomes. Administration of 10 μg of DTA (alone) did not show any effects in the mice (data not shown). These characteristics contrast with those of other low molecular weight anti-cancer drugs, and also may contribute to the absence of side-effects.

Although various methods have been developed to introduce foreign genetic materials into cells, only a few techniques have been developed to deliver proteins into the cytoplasm. The fusogenic liposomes in our system can deliver various kinds of proteins directly and efficiently into the cytoplasm because they fuse with the cell membrane through the mechanism of Sendai virus infection (Nakanishi *et al.*, 1985). The efficiency of fusogenic liposomes in introducing their contents into the cells is the same as that of infection with intact Sendai virus (Nakanishi *et al.*, 1995). Huang *et al.* reported that pH-sensitive immunoliposomes can deliver encapsulated DTA into the cytoplasm (Wang and Huang, 1989; Collins *et al.*, 1990; Litzinger and Huang, 1992; Tari *et al.*, 1994). However, these pH-sensitive immunoliposomes showed protein synthesis inhibition of only 50–70% at maximum, and could not kill all the cells (Collins *et al.*, 1990; Tari *et al.*, 1990). Judging from the concentration of DTA required to kill the cells, our system is far more efficient. This superior efficiency in protein delivery is another important aspect of possible cancer therapy using DTA.

The results described herein are a first attempt to examine the potential activity of fusogenic liposomes in cancer therapy. Fusogenic liposomes containing DTA may be a potential new therapeutic approach in the treatment of ascitic tumours and may also be effective in the local treatment of solid tumours and the treatment of malignant pleural effusion.

**Acknowledgements**
Hiroyuki Mizuguchi is a Research Fellow of the Japan Society for the Promotion of Science.

### References

CARROLL SF AND COLLIER RJ. (1988). Diphtheria toxin:quantification and assay. *Methods Enzymol.*, **165**, 218–225.

CASELLAS P, RAVEL S, BOURRIE BJP, DEROCQ J-M, JANSEN FK, LAURENT G AND GROS P. (1988). T-lymphocyte killing by T101-ricin A-chain immunotoxin: pH-dependent potentiation with lysosomotropic amines. *Blood*, **72**, 1197–1202.

COLLINS D, LITZINGER DC AND HUANG L. (1990). Structural and functional comparisons of pH-sensitive liposomes composed of phosphatidylethanolamine and three different diacylsuccinylglycerols. *Biochim. Biophys. Acta*, **1025**, 234–242.

COLOMBATTI M, ARCIPRETE LD, CHIGNOLA R AND TRIDENTE G. (1990). Carrier protein–monensin conjugates:enhancement of immunotoxin cytotoxicity and potential in tumor treatment. *Cancer Res.*, **50**, 1385–1391.

CROOKE ST. (1979). Mitomycin C: an overview. In *Mitomycin C: Current Status and New Developments*, Carter SK and Crooke ST (eds) pp. 1–4. Academic Press: New York.

KATO K, NAKANISHI M, KANEDA Y, UCHIDA T AND OKADA Y. (1991*a*). Expression of Hepatitis B virus surface antigen in adult rat liver. *J. Biol. Chem.*, **266**, 3361–3364.

KATO K, KANEDA Y, SAKURAI M, NAKANISHI M AND OKADA Y. (1991*b*). Direct injection of Hepatitis B virus DNA into liver induced Hepatitis in adult rats. *J. Biol. Chem.*, **266**, 22071–22074.

LITZINGER DC AND HUANG L. (1992). Phosphatidylethanolamine liposomes: drug delivery, gene transfer and immunodiagnostic applications. *Biochim. Biophys. Acta*, **1113**, 201–227.

NAKANISHI M, UCHIDA T, SUGAWA H, ISHIURA M AND OKADA Y. (1985). Efficient introduction of contents of liposomes into cells using HVJ (Sendai virus). *Exp Cell Res.*, **159**, 399–409.

NAKANISHI M AND OKADA Y. (1993). Liposome-mediated introduction of macromolecules into living animal cells with the aid of HVJ (Sendai virus). In *Liposome Technology*, Gregoriadis G. (ed.) pp. 249–260. CRC Press: Florida.

NAKANISHI M, ASHIHARA K, SENDA T, KONDA T, KATO K AND MAYUMI T. (1995). Gene introduction into animal tissues. In *Trends and Future Perspectives in Peptide and Protein Drug Delivery*, Lee VHL, Hashida M and Mizushima Y (eds) pp. 337–349. Harwood Academic Publishers: The Netherlands.

SZOKA F AND PAPAHADJOPOULOS D. (1978). Procedure for preparation of liposomes with large internal aqueous space and high capture by reverse-phase evaporation. *Proc. Natl Acad. Sci. USA*, **75**, 4194–4198.

TAKAMATSU K, UCHIDA T AND OKADA Y. (1986). Specific purification of elongation factor 2 and isolation of its antibody. *Biochem. Biophys. Res. Commun.*, **134**, 1015–1021.

TARI AM, FULLER N, BONI LT, COLLINS D, RAND P AND HUANG L. (1994). Interactions of liposome bilayers composed of 1,2-diacyl-3-succinylglycerol with protons and divalent cations. *Biochim. Biophys. Acta*, **1192**, 253–262.

UCHIDA T. (1982). Diphtheria toxin; biological activity. In *Molecular Action of Toxins and Viruses*, Cohen P and Heyningen SV (eds) pp. 1–31. Elsevier Biomedical Press: Amsterdam.

UCHIDA T, KIM J, YAMAIZUMI M, MIYAKE Y AND OKADA Y. (1979). Reconstitution of lipid vesicles associated with HVJ (Sendai virus) spikes. Purification and some properties of vesicles containing non-toxic fragment A of diphtheria toxin. *J. Cell Biol.*, **80**, 10–20.

WANG C-Y AND HUANG L. (1989). Highly efficient DNA delivery mediated by pH-sensitive immunoliposomes. *Biochemistry*, **28**, 9508–9514.

WAWRZYNCZAK EJ. (1991). Systemic immunotoxin therapy of cancer: advances and prospects. *Br. J. Cancer*, **64**, 624–630.

WRIGHT S AND HUANG L. (1989). Antibody-directed liposomes as drug-delivery vehicles. *Adv. Drug Deliv. Rev.*, **3**, 343–389.

YAMAIZUMI M, MEKADA E, UCHIDA T AND OKADA Y. (1978). One molecule of diphtheria toxin fragment A introduced into a cell can kill the cell. *Cell*, **15**, 245–250.